# Are AD-Typical Regions the Convergence Point of Multiple Pathologies?

**DOI:** 10.3389/fnagi.2015.00042

**Published:** 2015-03-25

**Authors:** Sylvia Villeneuve, Miranka Wirth, Renaud La Joie

**Affiliations:** ^1^Helen Wills Neuroscience Institute, University of California, Berkeley, CA, USA

**Keywords:** aging, amyloid, tau, vascular, neurodegeneration, Alzheimer’s disease

The amyloid hypothesis proposes a serial model of causality whereby beta-amyloid (Aβ) initiates a cascade of negative events such as neurofibrillary tangle formation leading to neurodegeneration, and eventually clinical onset of Alzheimer’s disease (AD). While this hypothesis was mainly founded on genetic forms of AD observations, increasing results coming from Aβ imaging suggests that the reality for late-onset AD is more complex. Clearly, the disease develops in an older brain, where age-associated comorbid factors are more prevalent and therefore have a more significant influence on disease expression. Furthermore, it is well established that around one-third of cognitively normal older adults have abnormal Aβ accumulation in their brain (Aizenstein et al., 2008), indicating that Aβ alone might not be sufficient to lead to the clinical expression of late-onset AD. Most of the late-onset AD cases might therefore be the consequence of multi-factorial pathologies (Chételat, 2013).

Alzheimer’s disease is associated with a characteristic pattern of macroscopic neurodegeneration (that can be detected *in vivo* using MRI and FDG PET biomarkers) in limbic and heteromodal regions of the cerebral cortex, here referred to as AD-typical regions (Dickerson et al., 2009; Landau et al., 2009; Schroeter and Neumann, 2011; La Joie et al., 2012; Wirth et al., 2013a). In this opinion paper, we argue that multiple factors work together with Aβ to hasten neurodegeneration in these limbic and heteromodal brain regions. Specifically, we propose that brain regions typically found to be atrophied and/or hypometabolic in AD dementia are vulnerable to multiple, and at least partly independent, pathologies (e.g., Aβ, tau, and vascular factors) and therefore represent regions where the impact of these pathologies converges (Figure 1). We further suggest that some of these pathologies might interact (i.e., have a synergistic effect) in AD-typical regions and that most of Aβ-related neurodegeneration might in fact be the consequence of these interactions. This hypothesis would explain why some individuals show cognitive impairment with relatively low levels of Aβ, while others have very high levels of Aβ without cognitive deficits. We therefore suggest that even if Aβ might be a needed pathological feature of late-onset AD clinical expression, its harmful effect might depend on other pathological factors that could emerge independently. This viewpoint thus emphasizes the idea that multiple pathways can trigger AD-typical atrophy/hypometabolism and contribute to the clinical expression of AD. While these pathways can be due to AD or non-AD factors, the convergence of these “other” pathways with β-amyloidosis might be needed for the development of cognitive deficits (Wirth et al., 2013a; Mormino et al., 2014) and clinical progression to dementia (Knopman et al., 2012).

**Figure 1:**
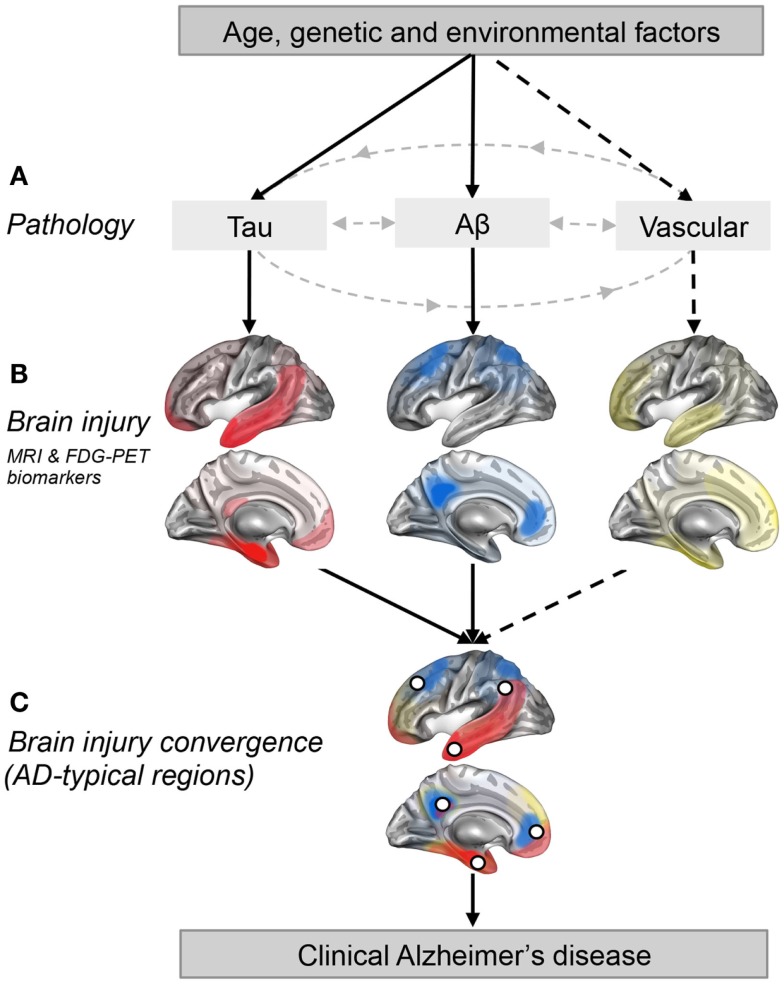
**Regional convergence of different pathologies (and their impact) involved in the clinical expression of late-onset Alzheimer’s disease**. **(A)** Solid lines represent pathologies that are needed to develop the clinical onset of Alzheimer’s disease (AD), while the dotted line represent a pathological factor that is not needed but, if present increases the risk of developing AD. **(B)** The pattern of brain injury associated with tau, Aβ, and vascular pathologies is represented by a schematic illustration based on current literature (Braak and Braak, 1991; Whitwell et al., 2008; Debette et al., 2011; La Joie et al., 2012; Villeneuve et al., 2014). **(C)** White dots represent brain regions where at least two pathologies are converging. These white dots also represent brain regions that are typically found to be atrophied and/or hypometabolic in individuals with AD (Dickerson et al., 2009; Landau et al., 2009; Schroeter and Neumann, 2011; Wirth et al., 2013a), and when atrophied in cognitively normal older adults, they increase the risk of progression to AD (Dickerson et al., 2009).

It became evident in the past years that Aβ is not the only factor driving neurodegeneration in AD-typical regions (Fjell et al., 2013; Wirth et al., 2013b). This suggests that other factors may work with Aβ to cause the brain changes typically found in patients with AD. Neurofibrillary tangles, which consist of microtubule-associated protein tau, are the other hallmark of AD. Even though the amyloid cascade hypothesis postulates that Aβ leads to tau pathology, neurofibrillary pathology can develop independently and prior to Aβ accumulation (Spillantini and Goedert, 2013). “Primary age-related tauopathy” (PART) has recently been proposed to describe a pathology that is commonly observed in the brains of older individuals (Crary et al., 2014). From this perspective, Aβ and tau can increase the risk of AD via independent mechanisms that work together to induce synaptic and neuronal loss (Small and Duff, 2008). This idea does not exclude the possibility that Aβ can induce tau pathology, rather it suggests that tau pathology can occur independently of Aβ and that individuals who have PART might be more vulnerable to Aβ if the latter starts to accumulate.

Recent work has proposed that Aβ and tau, measured by CSF levels, interact to trigger neurodegeneration in AD-typical regions such as the temporoparietal associative cortex (Fortea et al., 2014) and the entorhinal cortex (Desikan et al., 2014). Furthermore, medial frontal thinning associated with CSF p-tau seems to be present only in subjects with abnormal levels of Aβ (Fortea et al., 2014). Based on these interactions and because tau pathologies preferentially affect the temporal lobe (Braak and Braak, 1991; Whitwell et al., 2008), we suggest that temporoparietal AD-typical regions represent points of convergence between Aβ and tau pathologies (Figures 1B,C). Even if neocortical association areas are not primarily affected by tau accumulation (or its impact), they represent key regions where Aβ accumulates, and are probably one of the first regions were both pathologies meet (since the transentorhinal cortex is mainly spared from Aβ). This point of convergence of both pathologies might be what triggers their synergetic impact on brain integrity.

Cerebrovascular disease (e.g., cerebral microbleeds, white matter lesions, infarcts) and vascular risk factors (e.g., hypertension, dyslipidemia, and diabetes) are prevalent in older individuals and are known to increase the risk of AD (Prins and Scheltens, 2015). Even if such factors are not needed for the development of AD, they seem to increase the risk of AD by targeting brain regions vulnerable to AD (Wirth et al., 2013b; Villeneuve et al., 2014). Neurodegenerative abnormalities in cortical thickness and glucose metabolism in AD-typical regions have, for instance, been associated with white matter lesions in cognitively normal older adults (Wirth et al., 2013b). While white matter lesions do not seem to interact with Aβ to potentiate neurodegeneration (Haight et al., 2013), they nevertheless appear to have an additive impact on brain integrity (Chui et al., 2012). Vascular risk factors, particularly low levels of HDL cholesterol, have in turn been found to interact with Aβ to reduce cortical thickness in AD-typical regions such as the precuneus, the temporoparietal associative cortex, and the superior and middle frontal cortices (Villeneuve et al., 2014). This interaction suggests that the impact of Aβ on cortical thickness in AD-typical regions is potentiated in the presence of vascular risk (and/or vice versa). While Aβ deposition (La Joie et al., 2012) and its impact on neurodegeneration (Chételat et al., 2010; Villeneuve et al., 2014) is predominant in frontal and posterior association areas in individuals with cognitive impairments, vascular pathologies preferentially affect the frontal and temporal lobes (Jagust, 2013; Thal et al., 2014; Villeneuve et al., 2014). Therefore, brain regions such as the frontal lobe or the temporoparietal cortex represent converging points between Aβ and vascular pathologies. Even if evidence is missing for an interaction between tau and vascular factors in AD-typical regions, such interaction cannot be excluded and temporal regions such as the hippocampus are known to be vulnerable to both tau and vascular pathologies (Braak and Braak, 1991; Debette et al., 2011). Therefore, some AD-typical regions might also represent points of convergence between tau and vascular pathologies.

Figure 1 is a schematic illustration of our main hypothesis and does not represent real data. In Figure 1A, we propose that both the Aβ and tau pathways are needed for AD clinical expression while other pathways such as the vascular pathway (related to vascular risk factors and/or vascular brain injuries) are not. Figure 1B represents brain regions most affected (injured) by each pathology in cognitively impaired individuals (Braak and Braak, 1991; Whitwell et al., 2008; Debette et al., 2011; La Joie et al., 2012; Villeneuve et al., 2014). It is important to stress that these maps probably vary from one individual to another as the effects of a pathology on brain integrity likely depend on the length of time the pathology has been present, the amount of pathology, the location of the pathology (particularly true for vascular brain injuries which can be more focal than tau and Aβ) as well as other genetic and environmental factors that could influence vulnerability to each pathological process. Also, it is extremely difficult to isolate the degree of importance of a single pathology since different pathologies frequently occur together and probably interact, as argued in this opinion paper. The AD-typical regions, shown as white dots in Figure 1C, represent brain regions that are typically atrophied and/or hypometabolic in individuals with AD (Dickerson et al., 2009; Landau et al., 2009; Schroeter and Neumann, 2011; Wirth et al., 2013a). In this opinion paper, we further suggest that they represent the point of convergence of multiple pathologies, as well as brain regions where pathologies might have a synergistic effect.

This viewpoint does not explain how one pathology may potentiate the other or why some brain regions might be more vulnerable to multiple pathologies (Seeley et al., 2009; Buckner and Krienen, 2013; Jagust, 2013). Rather, it stresses the importance of considering late-onset AD as a multi-factorial process and questions the notion that Aβ-negative individuals presenting atrophy or hypometabolism in AD-typical regions are at low risk of AD, especially if they are close to the threshold for Aβ-positivity. Indeed, if other pathologies interact with Aβ, low (or subthreshold) Aβ levels could be sufficient to be harmful in the presence of these other pathologies. In conclusion, while Aβ may be needed to develop the clinical symptoms associated with AD, other factors might work together with Aβ to promote brain injury in AD-typical regions.

## Glossary

AD-typical regions = limbic and heteromodal regions of the cerebral cortex typically found to be atrophied and/or hypometabolic in patients with dementia due to AD.

## Conflict of Interest Statement

The authors declare that the research was conducted in the absence of any commercial or financial relationships that could be construed as a potential conflict of interest.
